# Economic burden and health related quality of life of ultra-rare Gaucher disease in China

**DOI:** 10.1186/s13023-021-01963-6

**Published:** 2021-08-11

**Authors:** Xinye Qi, Jiao Xu, Linghan Shan, Ye Li, Yu Cui, Huan Liu, Kexin Wang, Lijun Gao, Zheng Kang, Qunhong Wu

**Affiliations:** 1 Department of Health Policy, Health Management College, Harbin Medical University, No. 157, Baojian 21 Road, Nangang District, Harbin, 150081 Heilongjiang Province China; 2 Department of Social Medicine, School of Public Health, Harbin Medical University, Harbin, Heilongjiang China

**Keywords:** Gaucher disease, Health service utilization, Cost of illness, Health status, Caregiver, Diagnoses

## Abstract

**Background:**

The diagnosis and health care of patients with rare diseases present a tremendous challenge worldwide. This study described the health care service utilization through participants’ perspective and estimated the cost of illness (COI), and patients with Gaucher disease (GD)’s/caregivers’ health-related quality of life in China.

**Method:**

An online retrospective survey of patients with GD and their caregivers was conducted during May–June 2018. Socio-demographic, health service utilization, disease-related expenses, social support, sleep quality (Pittsburgh Sleep Quality Index [PSQI]), and the Short Form Health Survey (SF-36) were investigated. Using self-reported information, we estimated the annual COI, including direct healthcare, direct non-healthcare, and indirect costs.

**Results:**

Forty patients and their 49 caregivers were surveyed. The patients’ onset age of GD was 9.3 ± 10.9; their disease course was 3.5 ± 3.1 years. 21 (42.9%) patients had ≥ 2 caregivers, but 35 (71.4%) caregivers reported have no experience as a caregiver. 79.6% caregivers have stopped working, and 87.8% changed weekly working schedule. Before final diagnosis, patients visited 3.9 ± 3.1 (max = 20) hospitals and took 1.2 ± 1.7 (max = 6.6) years for confirmed diagnosis. On average, 5.0 ± 9.6 misdiagnoses occurred, and the per-patient diagnoses cost was USD ($) 7576. After GD confirmation, 8 (16.3%) patients received no treatment, 40 (81.6%) received pharmacotherapy, 10 (20.4%) received surgery, 38 (77.6%) received outpatient service (8.8 ± 9.1 times/annually), and 37 (77.5%) received inpatient service (4.0 ± 3.5 times/annually). Annual per-patient COI was USD ($) 49,925 (95% confidence interval: 29,178, 70,672). Average direct healthcare cost was $41,816, including pharmaceutical ($29,908), inpatient ($7,451), and outpatient ($1,838). Productivity loss per-caregiver was $1,980, and their Zarit Burden Inventory score was moderate-severe (48.6 ± 19.6). Both patients/caregivers reported lower social support (32.4 ± 7.4, 34.9 ± 7.6), two times higher PSQI (7.9 ± 2.9, 8.7 ± 3.6), and half lower SF-36 (41.3 ± 18.6, 46.5 ± 19.3) than those reported for healthy Chinese individuals.

**Conclusions:**

The high misdiagnosis rate, together with delayed diagnosis, substantial costs, and deteriorated health-related quality of life of GD patients as well as their heavy care burden, calls for extreme attention from policymakers in China. Further efforts of government and society are urgently demanded, including pharmaceutical reimbursement, screening newborns, developing precise diagnostic tools, and training doctors.

**Supplementary Information:**

The online version contains supplementary material available at 10.1186/s13023-021-01963-6.

## Background

Gaucher disease (GD), an ultra-rare multi-organ involvement, inherited recessive enzyme deficiency [1, 2], is slowly progressing. Its standardized incidence and prevalence among the general population worldwide vary from 0.30 to 5.80 per 100,000 population, respectively; however, the initial estimate was 0.25 to 1.24 per 100,000 population [3, 4] with hundreds of patients diagnosed with GD in China. Accompanying symptoms of GD include anemia, thrombocytopenia, splenomegaly, hepatomegaly, and bone involvement [5], which often cause significant mpairs to their health and the quality of life. And once diagnosed, it ought to be managed with enzyme replacement therapy [6, 7], but results in enormous expenses.

Currently, the effective therapy for GD patients, such as enzyme replacement therapy and substrate reduction therapy, which must be administered continually, are extremely costly for patients and caregivers [8, 9]. Further, caregivers may experience workdays loss, absenteeism, and job changes while caring for their patients [10]. Measuring the economic cost of illness can provide better information to policymakers to develop more targeted interventions to GD at various levels of the health care system [11]. Measuring the economic and care burdens of diseases to the society will facilitate the prioritized healthcare policies and strategies [1]. However, evidence on the financial burden (e.g., direct healthcare costs, direct non-healthcare costs, and indirect costs) associated with GD in China is limited [12, 13]. Extensive health care utilization and frequent medical care and expenses are associated with the economic burden of patients and caregivers [14]. Hence, it is essential to identify costs and health-related quality of life of GD patients/caregivers in China [15].

The World Health Organization has emphasized the need for urgent action to diagnose and treat people with rare diseases [16]. However, the diagnosis and treatment of rare diseases in China are still at a preliminary stage. Definitive GD diagnosis in China is difficult, especially with the high phenotypic heterogeneity of GD [17]. Several studies have explored the length of time required for diagnosing rare diseases [18, 19]. A long duration is often required before a correct diagnosis or treatment, which implies considerable discomfort for the patients with rare diseases, such as GD, and their families. To the best of our knowledge, few studies have focused on health service utilization and costs of GD, including the diagnosis [20].

This study aimed to describe the health care service utilization through participants’ perspective, estimated the cost of illness, health-related quality of life of patients with GD, and the quality of life of their caregivers.

## Methods

### Development of the survey

An online retrospective survey of patients with GD and their caregivers was conducted for data collection. The inclusion criteria for patients were as follows: (i) a diagnosis of GD by a physician before the study; (ii) with a caregiver who was familiar with the whole treatment process of them; (iii) if the patient was under 15, with a voluntary caregiver to help answer all the real information of them. The inclusion criteria for the caregivers included that he/she was: (i) the primary caregiver ; (ii) was thoroughly familiar with the patient's disease; (iii) accompany patients every visit to the treatment; and (vi) was able to understand the content of the questionnaire.

The China Gaucher disease family exchange group (C-GDFEG) is currently the largest GD information exchange platform in China. The questionnaire, including the patient part and the caregiver part, was distributed simultaneously to eligible patients with GD and their caregivers. In the case of patients < 15 years or patients who are dependent partly or on full-time care, the patient part asked whether the patient can answer independently, and questions were set up in two ways (et al., “Birthdate of you” or “Birthdate of the patient”). The patient part was answered by themselves or with their caregivers’ help if they can understand questions; if not, patients’ information will be answered only by their caregivers.

### Questionnaire design and data collection

From the literature review, we identified key areas that were both important for the patients and families, and essential for the health authorities and service provision planning. The online self- administered questionnaire for the patients with GD, their caregivers, and their households comprised the following sections: (i) socio-demographic characteristics; (i) health service utilization ; (ii) cost of illness; (iii) social support; (iv) sleep quality; (v) care burden; and (vi) self-reported health (using SF-36).

### Definition and measurements

#### Socio-demographic characteristics

The patients’ socio-demographic characteristics included age, sex, current residence, mean age at onset, disease duration, type of GD, family history of GD, and caregivers’ number. Socio-demographic characteristics of the caregivers included the daily care time, experience of being a caregiver, stopped working because of patients’ GD, change of weekly working scheme because of patient’s GD, and members with chronic disease > 60/ < 5 years of age. Besides, whether living on minimum subsistence allowance or relatives' relief or not was also reported.

#### Health service utilization and caregivers’ perception of GD treatment

The health service utilization data were collected in this study to assess patients’ diagnosis and treatment status. Health service utilization covering the 12 months health service utilization of patients with GD surveyed by self-designed questions, including the following: (i) the original reason to see the doctors; (ii) symptom; (iii) types of medical institutions frequently visited; (iv) the number of hospitals visited for GD diagnosis; (v) total cost of GD diagnosis; (vi) length before the final GD diagnosis was confirmed; (vii) misdiagnoses ; (viii) the number of misdiagnoses ; (ix) therapy after GD diagnosis; (x) outpatient/inpatient visit for GD; (xi) traffic time; and (xii) surgery/pharmacologic therapy after GD diagnosis.

Additionally, caregivers’ perceptions, “the availability and affordability of therapeutic drugs,” “difficulties during GD treatment,” “knowledge of GD,” and “difficulties to get diagnosis information” were also investigated.

#### Cost of illness

We retrospectively evaluated the total cost of GD, comprising direct healthcare, direct non-healthcare, and indirect costs for patients and their families. Costs comprised the following components: *Direct healthcare costs*: (i) daily outpatient/inpatient treatment cost; (ii) laboratory and diagnostic costs; (iii) imported/domestic drugs’ costs; (iv) formal care cost; (v) other direct outpatient/inpatient medical costs; *Direct non-healthcare costs*: (vi) outpatient/inpatient costs for accommodation/transportation/meals; (vii) artificial nutrition costs; (viii) other direct non-healthcare costs; and *Indirect costs*: (ix) daily lost wages of caregivers.

Respondents were also asked to recall the use of resources: (i) outpatient/inpatient visit frequencies (consultations ) annually ; (ii) the average days for each outpatient/inpatient visit; and (iii) total days of lost wages annually. The resources used were multiplied by the corresponding unit cost to estimate the annual cost per patient, and the monetary values of the unit prices were reported in USD [$, Purchasing Power Parities in the study years of OECD National Accounts Statistics (2018): https://data.oecd.org/conversion/purchasing-power-parities -ppp.htm#indicator-chart.]

#### Zarit Burden Inventory

The subjective burden of the caregivers was measured by ZBI (22-item version), with item scores ranging from 0 (never) to 4 (nearly always). The total score ranges from 0 to 88, and scores < 21 correspond to little or no burden while scores > 61 correspond to severe burden [21].

#### Social Support Rating Scale

SSRS comprises 10 items and includes three subscales: subjective support (4 items), objective support (3 items), and utilization of support (3 items). The total SSRS score ranges from 12 to 66 points, and higher scores indicate a higher level of social support [22].

#### Pittsburgh Sleep Quality Index

Sleep quality of individuals in 1 month was measured using the PSQI (comprises 19 items and 7 dimensions), which includes subjective sleep quality, sleep onset latency, total sleep duration, sleep efficiency, sleep disturbances, use of sleep medication, and daytime dysfunction. The sub-total of the scores of each dimension ranges from 0 to 3, and the maximum score is 21. The cut-off score for PSQI-defined cases of poor sleep quality is ≥ 6. A Chinese PSQI version has been validated with adequate reliability [23].

#### The medical outcomes study 36-item Short Form

Quality of life was measured by the SF-36 comprising 36 items, including the physical and mental component scores [24]. Subscale scores are then transformed from the normal scale to a 0–100 standardized score scale, with higher scores indicating more positive health status and a better health-related quality of life.

### Statistical analyses

Descriptive analysis was conducted to evaluate the social demographic characteristics, the status of health service utilization, cost of illness, and health-related quality of life. Continuous variables were presented as means ± SDs, and categorical variables were presented as absolute and relative frequencies. The 2.5 and 97.5% quantiles of the bootstrap distribution were used as the limits of the 95% CI. Subgroup comparisons of health-related quality of life and COI were conducted using student t-tests for two groups and one-way ANOVA tests for three or more groups. Correlation analysis were used to continuous variables. All statisitical analyses were conducted using SAS software, version 9.4 (SAS Institute, North Carolina, US).

## Results

### Socio-Demographic characteristics

From May to June 2018, we contacted a total of 98 families of patients with GD in C-GDFEG before conducting this survey. A total of eligible 51 (52.04%) patients enrolled in this study. Both the patient part and the caregiver part completely answered was considered as a valid questionnaire. Of them, 49 (validity rate 96.08%) patients with GD and their caregivers (n = 49) completed the questionnaire were included in this study. The contact details of the researchers were also sent to the participants for information and further communication.

Characteristics of enrolled patients with GD, families, and caregivers are shown in Table 1, which shown GD often occur in child and adolescents.

**Table 1:** Characteristics of enrolled patients with Gaucher disease

Items	Sample (n)	Proportion (%)
Sex		
Male	25	51.0
Female	24	49.0
Age group (mean age at survey) in years		
0–4	19	38.9
5–7	5	10.2
8–12	12	24.5
13–14	2	4.1
≥ 15	11	22.5
Age at onset, years		
Mean ± SD	9.3 ± 10.69	
Max	44	
Min	1	
Disease course from symptom onset, days		
Mean ± SD	3.5 ± 3.1	
Max	12.1	
Min	0.3	
Median	2.0	
Types of Gaucher disease (GD)		
Type I	20	40.8
Type II	5	10.2
Type III	4	8.2
Not clear	20	40.8
Number of caregivers		
1	28	57.1
≥ 2	21	42.9

*SD* standard deviation

Among patients’ families (see in Additional file 1: Table 1), 18.4% had a family history of GD, and 85.7% had family members aged > 60 years. We have measured the daily care time of care by a closed-form problem with four options (1 =  < 6 h, 2 = 6–12 h, 3 = 12–18 h, 4 = 18–24 h); therefore, we are unable to provide the mean daily care time of care. Among the 49 caregivers, 81.6% reported caring for the patients for > 6 h daily, and 71.4% having no caregiving experience. Further, 79.6% and 87.8% of caregivers stopped working and absenteeism. 79.6% changed their weekly working schedule. The Zarit Burden Inventory (ZBI) score, indicating the burden of the caregivers, was from moderate to severe (48.57 ± 19.57).

### Health service utilization

The profile of health service utilization of patients with GD is shown in Additional file 1: Table 2. Patients’ original reasons for visiting the doctors included enlarged liver and spleen (71.4%), normocytic anemia (36.7%), dyskinesia (12.2%), and osteopenia (8.2%). Among all surveyed patients, the two most frequent symptoms were hepatosplenomegaly (85.7%) and normocytic anemia (73.5%). Many patients (43.5%) reported that they had to travel to the tertiary hospitals in other provinces to get confirmed diagnosed. Among 49 patients, 77.6% received outpatient service, and 77.5% received inpatient service.

Hardly recognized by many doctors, the patients visited 3.9 ± 3.1 hospitals, and it took 1.2 ± 1.7 (median = 0.4, max = 6.6) years for the diagnosis to be confirmed. The total cost for GD diagnosis was USD ($) 7575.8 (median); 59.2% of patients experienced ≥ 1 misdiagnosis, with a mean of 5.0 (SD = 9.6, median = 2.0). Before the correct diagnosis, most patients were misdiagnosed with unexplained splenomegaly (65.5%), hypersplenism (44.8%), and hemophilia (10.2%). Eight (16.3%) patients received no health service following GD diagnosis. Ten patients underwent surgery (20.4%; splenectomy = 9; hematopoietic stem cell transplantation = 1). The remaining 40 (81.6%) received pharmacotherapy, among whom 14 (35.0%) had not used medications as prescribed, 27 (67.5%) reported using imported drugs, 9 took imiglucerase (the medicine used treatment of GD), and 5 were still using imiglucerase from the onset of GD diagnosis; all 49 (100%) agreed that the high price of medicine led to the failure of compliance with the doctor's prescription. Furthermore, 36 (90.0%) reported purchasing their medications at medical institutions or pharmacies, while 31 (71.5%) purchase from overseas.

Additional file 1: Table 1 shows the caregivers’ perception of GD treatment. Most (89.8%) caregivers perceived that the availability of therapeutic drugs was problematic. All (100%) caregivers reported their inability to afford therapeutic drugs. 48 (98.0%) Caregivers reported have no knowledge of GD. Most (96.0%) caregivers felt difficulty in obtaining diagnosis information. The top two problems confronting the caregivers during GD treatment were “high treatment cost” (95.8%) and “far away from hospitals” (75.0%). Up to 79.6% of 49 caregivers were extremely unconfident about future treatment.

### Cost of illness

The cost of illness of patients with GD is shown in Table 2. In total, the mean annual economic burden of GD was estimated at $48,771. The mean direct healthcare costs of GD estimated at $41,816.2 (85.7% of total costs) mainly comprised of taking medicine at home (61.3% of total costs), inpatient treatment (15.3%), outpatient treatment (3.8%), and medical tests (3.4%).

**Table 2:** Average annual cost of illness due to Gaucher disease (GD) in China ($, n = 41)

	Mean annual costs	95% CI		Ratio of COI (%)
		Low limit	Upper limit	
Direct healthcare cost	41,816.2 ± 65,745.4	21,064.4	62,568.0	85.74
Outpatient medical treatment	1838.1 ± 3029.4	881.9	2794.4	3.77
Inpatient medical treatment	7451.2 ± 10,392.2	4171.1	10,731.4	15.28
Pharmaceutical treatment	29,907.6 ± 63,861.1	9750.6	50,064.7	61.32
Medical tests	1675.9 ± 3207.2	663.6	2688.2	3.44
Formal care	580.6 ± 817.7	322.5	838.7	1.19
Other cost/expenditures	362.7 ± 654.3	156.2	569.3	0.74
Other cost/expenditures of outpatient	88.1 ± 159.7	37.7	138.5	0.18
Other cost/expenditures of inpatient	274.6 ± 633.0	74.8	474.5	0.56
Direct non-healthcare cost	4974.4 ± 4675.9	3498.5	6450.3	10.20
Accommodation	1245.0 ± 1711.2	704.9	1785.1	2.55
Accommodation of outpatient	440.3 ± 597.8	251.6	629.0	0.90
Accommodation of inpatient	804.7 ± 1562.6	311.5	1297.9	1.65
Transportation	819.9 ± 841.5	554.3	1085.5	1.68
Transportation of outpatient	581.8 ± 685.9	365.3	798.3	1.19
Transportation of inpatient	238.0 ± 376.3	119.3	356.8	0.49
Meals	749.8 ± 874.9	473.7	1026.0	1.54
Meals of outpatient	277.3 ± 356.2	164.8	389.7	0.57
Meals of inpatient	472.6 ± 740.1	239.0	706.2	0.97
Costs for artificial nutrition	1340.0 ± 2796.6	457.3	2222.7	2.75
Other cost/expenditures	819.7 ± 929.0	526.4	1112.9	1.68
Direct cost	46,790.6 ± 65,714.2	26,048.6	67,532.5	95.94
Indirect cost				
Productivity loss to caregiver due to caring	1980.4 ± 1962.8	1360.9	2600.0	4.06
Cost of illness (COI)	48,771.0 ± 65,616.8	28,059.8	69,482.2	100.00

Because of rounding, percentage might not add up to exactly 100%

COI = cost of illness; COI is the annual per-patient in this study

^*^The medical costs were converted to US Dollar using Purchasing Power Parities (PPP) in the study years (2018) (OECD National Accounts Statistics: PPP https://data.oecd.org/conversion/purchasing-power-parities -ppp.htm#indicator-chart)

The average annual direct non-healthcare costs added up to $4,974.4 (10.2% of total costs). With both direct healthcare and non-healthcare costs, the annual direct cost was estimated at $46,790.6. The indirect costs for GD primarily originated from the productivity loss to the caregivers while caring for patients with GD (4.1% of total costs).

### Social support and health-related quality of life of patients and their caregivers

The total Social Support Rating Scale **(**SSRS), Pittsburgh Sleep Quality Index (PSQI), and Short Form Health Survey (SF-36) scores for patients with GD, their caregivers, and norms of healthy Chinese people are shown in Table 3. Patients with GD and their caregivers reported mean SSRS scores of 25.3 ± 5.7 and 26.1 ± 5.6, respectively. Both were remarkably lower than the SSRS score (40.5 ± 6.8) for healthy Chinese people (all p-value < 0.05). The mean PSQI scores for patients and caregivers scores were 7.9 ± 2.9 and 8.7 ± 3.6, respectively. Patients with GD and their caregivers reported two times higher PSQI scores than the normal values for healthy Chinese people (3.9 ± 2.5) (all p-value < 0.05). Overall, 16 (32.7%) of caregivers rated sleep problems using PSQI dimensions. All the seven PSQI dimension scores for the caregivers were higher than the normal values for healthy Chinese people (all p-value < 0.05).

**Table 3:** Psychosocial burden of Gaucher disease on the caregivers

	Patients (n = 30)	Caregivers (n = 49)	Norm of healthy Chinese people	P^a^	p^b^
SSRS^c^ Maximum score, 40 points					
Objective support	8.2 ± 2.5	8.2 ± 2.3	9.1 ± 2.9	0.059	0.009
Subjective support	17.6 ± 5.0	19.9 ± 4.9	23.5 ± 4.3	< 0.001	< 0.001
Utilization of support	6.6 ± 1.8	6.7 ± 1.9	7.8 ± 2.0	0.001	< 0.001
Mean ± SD	32.4 ± 7.4	34.9 ± 7.6	40.5 ± 6.8	< 0.001	< 0.001
PSQI ^d^ Maximum score, 21 points					
Not good	6 (20.0)	16 (32.7)			
Good	24 (80.0)	33 (67.3)			
Mean ± SD	7.9 ± 2.9	8.7 ± 3.6	3.9 ± 2.5	< 0.001	< 0.001
Subjective sleep quality	1.57 ± 0.97	1.71 ± 0.89	0.63 ± 0.68	< 0.001	< 0.001
Sleep latency	1.77 ± 0.77	1.59 ± 0.91	0.70 ± 0.86	< 0.001	< 0.001
Sleep duration	0.87 ± 1.01	1.24 ± 0.97	0.70 ± 0.58	< 0.001	< 0.001
Habitual sleep efficiency	0.27 ± 0.69	0.63 ± 0.86	0.15 ± 0.47	< 0.001	< 0.001
Step disturbance	1.73 ± 0.52	1.61 ± 0.57	0.90 ± 0.44	< 0.001	< 0.001
Use of sleeping medication	0.00	0.08 ± 0.34	0.06 ± 0.24	–	< 0.001
Daytime dysfunction	1.67 ± 1.03	1.82 ± 1.11	0.73 ± 0.83	< 0.001	< 0.001
SF-36^e, f^ Maximum score, 100 points for each item					
Total score	41.3 ± 18.6	46.5 ± 19.3			
Physical Component Summary (PCS)	39.4 ± 22.4	47.6 ± 23.0	77.5 ± 16.0	< 0.001	< 0.001
Mental Component Summary (MCS)	43.2 ± 16.1	45.4 ± 20.4	71.3 ± 17.9	< 0.001	< 0.001
Physical functioning (PF)	63.2 ± 20.2	63.8 ± 30.2	87.9 ± 17.0	< 0.001	< 0.001
Role physical (RP)	13.6 ± 32.3	27.6 ± 43.1	77.5 ± 34.9	< 0.001	< 0.001
Bodily pain (BP)	51.8 ± 23.3	60.3 ± 26.3	82.2 ± 17.0	< 0.001	< 0.001
General health (GH)	29.0 ± 25.9	38.9 ± 21.9	62.5 ± 17.9	< 0.001	< 0.001
Vitality (VT)	42.7 ± 16.5	45.3 ± 18.6	68.2 ± 17.6	< 0.001	< 0.001
Social functioning (SF)	71.6 ± 26.9	67.6 ± 30.0	80.7 ± 20.0	0.013	0.004
Role emotional (RE)	6.1 ± 20.1	20.4 ± 37.2	67.9 ± 39.4	< 0.001	< 0.001
Mental health (MH)	52.4 ± 17.1	48.3 ± 16.0	68.5 ± 16.9	< 0.001	< 0.001

SSRS = Social Support Rating Scale; PSQI = Pittsburgh Sleep Quality Index; SF-36 = Medical Outcomes Study 36-item Short Form; SD = standard deviation

^a^Patients vs. normal values for healthy Chinese

^b^Caregivers vs. normal values for healthy Chinese

^c^N = 3342 [72]

^d^N = 112 [73]

^e^N = 17,754 [74]

^f^N of patients ≥ 15 is 11

Besides, the health-related quality of life of the patients with GD and caregivers was assessed. Patients (39.4 ± 22.4) and caregivers (47.6 ± 23.0) reported a half lower Physical Component Summary than the normal values for healthy Chinese people (77.5 ± 16.0, all p-value < 0.05). Further, Mental Component Summary in both patients (43.2 ± 16.1) and caregivers (45.4 ± 20.4) was significantly half lower than the normal values in healthy Chinese people (71.3 ± 17.9, all p-value < 0.05).

The COI of GD patients with splenectomy surgery is significantly different with GD patients without (*F* = 4.634, *P* = 0.017), and GD patients taking medication as prescribed is significantly different with GD patients without (*F* = 6.454,* P* = 0.003). Duration of GD final confirmed diagnosis (*r* = 0.458, *p* = 0.003) and the difficulty to get diagnose (*r* = 0.388, *p* = 0.012) are positively relative to the COI of GD patients (Additional file 1: Table 4).

The quality of life of GD patients with bone pain or other bone symptoms is significantly different with non-bone symptoms (*F* = 4.984, *p* = 0.053). The health condition of GD patients (*r* = 0.760, *p* = 0.007) is positive relative to the quality of life of GD patients (Additional file 1: Table 5).

The quality of life of caregivers caring for patients with different types of GD is significantly different (*F* = 3.528, *P* = 0.022). Patients with nervous system involvement symptoms are significantly different with non (*F* = 2.675,* P* = 0.001). Economic pressure (*r* = -− 0.438, *p* = 0.002) and difficulty in caring for GD patients (*r* = − 0.490, *p* < 0.001) are negatively correlated with caregivers’ health-related quality of life. Health condition of GD patients (*r* = 0.465, *p* = 0.001) and the objective support received by GD caregivers (*r* = 0.320, *p* = 0.025) are positively related to the quality of life of GD caregivers (Additional file 1: Table 6).

## Discussion

There is a dearth of evidence on the health service utilization and economic burden of rare disease patients with GD in China. In this study, we identified problems of patients with GD in their health service utilization, cost of illness, and health-related quality of life associated with GD in China. Families of patients with GD often experienced delays in diagnosis and misdiagnosis, enormous economic cost, and caregiving burden. Lack of social support often resulted in deteriorated health related quality of life and other major health issues which deserve great attention of society.

Misdiagnosis or delayed diagnosis is especially detrimental to patients [25]. Although several publications have acknowledged that diagnostic delays or misdiagnosis often occurs in obtaining a definitive GD diagnosis [26], this study further quantified the exact time delay and difficulty in obtaining the correct diagnosis that occurred in 79.6% surveyed patients. Studies indicated the process of continually seeking diagnosis was a traumatic experience for patients with rare diseases [27]. Davari M et al. have shown a patient misdiagnosed with GD had taken medications for ten years at the cost of $207,580 [1]. Thus, a rapid precise diagnosis of patients is essential for reducing unnecessary tests or costs [26, 28, 29], and preventing psychological stress for the family [30].

The main reason for the prolonged diagnostic delay most likely is insufficient knowledge on GD [31–33] by the medical community due to its low prevalence, extreme variations in clinical manifestations [28, 34], and severity of symptoms (e.g., bone pain/bone crisis, thrombocytopenia, and splenomegaly ) [20, 26, 28]. Nevertheless, a survey revealed that only one of five hematologist–oncologist considers GD in the differential diagnosis of patients with a history of anemia, thrombocytopenia, hepatomegaly, splenomegaly, and bone pain. In this study, 79.6% of patients had visited doctors with obvious symptoms (osteopenia, normocytic anemia, dyskinesia, and obviously enlarged liver) in whom GD was not diagnosed in a timely manner. Therefore, establishing an information center of rare disease symptoms [35] and providing training on diagnostic methods and appropriate clinical guidelines to medical doctors are essential to effectively support the early diagnosis of rare diseases. Besides, because most rare diseases are caused by DNA mutations or are recessive genetic diseases [36], family history and genetic screening could facilitate earlier diagnosis, while genetic testing can be used to confirm disease diagnosis [37]. Nine families reported a family history of the same disorder in this study, indicating a need for awareness of genetic disorders to highlight the importance and burden of genetic diseases. Therefore, newborn screening for disorders that meet the criteria for population screening [38], use of reliable diagnostic tools [1, 39–41], raising of awareness on the early signs/symptoms [42] or less severe manifestation [26], and initiation of therapy early for rare diseases [35], are essential.

In particular, long-term treatment with regular monitoring for GD can optimize outcomes [43], alleviate fewer exacerbations [1], and reduce the incidence of complications [44]. A Gaucher outcome survey involved 1209 patients with GD reported the overall treatment (1209, 73.4%) and different treatment rates in countries [the UK (119, 93.3%), Israel (536, 63.6%), the US (380, 82.6%), rest of the world (174, 69.5%)] [45]. In this study, 8 of the 49 patients (16.3%) were never treated, while 83.7% get treated, and 10 (20.4%) of patients undergone splenectomy, which was higher than previous research (8.7–13.3%) [2, 45]. Besides, other studies have also shown a significant proportion of patients (e.g., asymptomatic or mildly affected) never accessed medical attention or required treatment [46, 47]. In this study, even 20 (40.8%) of patients did not clear the type of their GD. Maaswinkel Mooij et al. [48] found that no treatment for type 1 GD with enzyme replacement therapy can worsen the condition at any age in non-Jewish patients [49]. Besides, 4 of 9 patients who took imiglucerase interrupted their treatment in this study. However, Drelichman et al. emphasized the need to avoid interruption of medical treatment of patients with GD because of recurrent organomegaly, growth delays, and skeletal manifestations [50]. Furthermore, a previous study identified the association of high immunoglobulin (Ig) A and IgG levels with long-term complications [51]. Therefore, long-term treatment and surveillance are required for improving the efficiency of GD management [52].

To estimate the annual cost of GD, we presented the core components of costs for health care services separately, from the payer’ perspectives, excluding patients’ out-of-pocket costs. The estimated annual per-patient cost of GD of $48,771 (95% confidence interval [CI]: $28,060, $69,482, which is 5.2 times of GDP per capita of China in 2018($9,377) [53]. The additional time spent by caregivers for providing care reflects an annual productivity loss of $1,980 per patient. In contrast, a recent analysis using the human capital approach (economic evaluation) showed high costs [e.g., Splenectomy ($13,698), Bone complication ($10,002), Multiple complications ($10,615), and Malignancy ($73,057)] that generated much higher indirect costs applying the lifetime horizon than ours (cost-of-illness) [54]. Besides, Dussen et al. have also analyzed the loss of production due to work-days off (absenteeism) and loss of production due to early retirement, whereas we calculated indirect costs with the actual wage of the caregivers. Besides, direct non-healthcare costs ($4974), comprised those for accommodation, transportation, meals, and other expenditures, which accounted for 10.6% of direct costs; this indicated a high burden of access to healthcare services. Moreover, patients with GD required frequent healthcare utilization resulting in an annual direct cost (direct healthcare/non-healthcare costs) of $46,791 per patient (95% CI: $26,049, $67,533); that is, 95.9% of total costs. In this study, the mean direct healthcare costs of $41,816 per patient was about 66.5 times of the average health expenditure per patient ($629) in China in 2018 [55]. The direct healthcare costs included those for pharmaceutical ($29,908, 61.3%), inpatient (15.3%), and outpatient treatments (3.8%). One study in Iran estimated the annual direct healthcare costs at $20,758 in 2014 and identified drug costs as 95.2% ($19,763) of direct healthcare costs [1]. These figures clearly show that the pharmaceutical cost encompasses most of the resources used by patients with GD. Recent research has also demonstrated that drug expenditures accounted for almost 90% of rare diseases’ health expenditures [15]. Therefore, further efforts are needed to address the issue of pharmaceutical treatment costs holistically [56].

However, both the availability and affordability of orphan drugs [57] for the treatment of rare diseases in China are low [13]. Poor accessibility to drugs is the most problematic issue for patients with rare disease in China [58]. In this study, 89.8% of patients perceived the availability of therapeutic drugs to be problematic. Furthermore, 35.0% of patients who received pharmaceutical treatment could not take the medication as prescribed. These figures demonstrate poor availability of rare drugs. Several hindering factors contributing to the low availability of orphan drugs in China include the low market availability of orphan drugs in China [13, 59]. Compared with the earliest launch time globally, the average delay in the market authorization of orphan drugs for rare diseases in China was 7.7–9 years [13, 59]. Imiglucerase received China’s marketing authorization in 2008 [13]. On May 22, 2018, the Chinese Government officially included GD on the first list of rare diseases [60]. With a low availability of < 30% [13], many Chinese patients currently pay out-of-pocket for international treatments that are currently unapproved in China [61]. Second, lack of research and development and supply incentive policies for orphan drugs are possible reasons leading to low market availability [61]. Under a market-oriented economy, due to a small market share of orphan drugs, most pharmaceutical manufacturers are unwilling to invest in research and development on the production of orphan drugs without an incentive policy [13]. Third, low public hospital availability of orphan drugs is another major issue both for the patients [62] and the Chinese government. In this study, 31 (77.5%) of patients reported purchasing medicine at pharmacies or overseas. Due to the unique market attributes (e.g., low market volume, low profit, low turnover rate, and high price) of orphan drugs, and as the reform of public hospitals in China stipulated (control the share of drug sales in total hospital revenue), hospitals have little incentive to stock and prescribe expensive orphan drugs [63]. Lastly, there are no public national or provincial networks for rare diseases or orphan drugs to share useful information about these diseases, e.g., treatment or supply information. Therefore, it is urgent to improve the availability of rare drugs, e.g., simplify the approval procedure for imported orphan drugs, increase research and development investment, formulate incentive policies, and establish information-sharing platforms.

Furthermore, to recoup research and development costs and for profit, orphan drug manufacturers often adopt a strategy of charging high prices [64]. Miglustat and imiglucerase, which are effective medications for treating GD, had annual costs of $116,800 and $140,200, respectively in 2004 [65], and the expenditure share was > 3% of per capita GDP [12]. A study focusing on seven selected rare diseases found that the affordability of treatment was relatively poor, with the health expenditure for GD equivalent to 69.34 years per urban resident’s income in 2014 [12]. In this study, all patients reported the unaffordability of therapeutic drugs. The COI of GD patients taking medication as prescribed ($68,759.5 ± $75,457.7) is higher than GD patients without ($17,277.7 ± $19,382.4). High prices discourage patients from taking their medicine as prescribed. But considering the benefits of prescribed medication for patient control, clinicians should encourage patients to take medication as directed. A few cities/provinces are active in creating rare disease lists with a high reimbursement at 80–95% [66]. However, due to the restrictive reimbursement caps, even with 5% out-of-pocket expenses, few drugs could be afforded by high-income urban residents [67]. The out-of-pocket costs are still unaffordable for many patients. Therefore, the low affordability of orphan drugs may be closely associated with many factors, e.g., high drug prices, lack of insurance coverage, low reimbursement rates, and low-income levels for Chinese residents [13]. Several measures should be taken to improve the affordability of orphan drugs in China, including (1) formulating and implementing incentive policies [64, 68] to promote the development and supply of generic drugs, and to control the price of orphan drugs [69, 70]; (2) developing an orphan drug reimbursement system to increase insurance coverage; and (3) developing government-supported programs for patients with rare disease.

Due to the delayed diagnosis, unavailability, and unaffordability of costly drugs, 79.6% of 49 patients with GD felt poorly confident about future treatment. Moreover, the total social support, sleep quality, and quality of life scores for patients with GD/caregivers were significantly lower than those for normal healthy Chinese people. In this study, the health-related quality of life of GD patients with bone pain or other bone symptoms (31.58 ± 5.54) is significantly lower than patients who have non-bone pain symptoms (52.95 ± 22.72). Caregivers of type 1 GD patients have the highest health-related quality of life (51.29 ± 20.31), followed by caregivers of type 3 GD patients (40.01 ± 16.65) and caregivers of type 2 GD patients have the worst quality of life (23.38 ± 11.62). The health-related quality of life of caregivers of GD patients with nervous system involvement symptom (29.29 ± 11.52) is significantly lower than caregivers of non-neural system involvement GD patients (50.95 ± 18.49). Clinicians should pay more attention to GD patients accompanied by skeletal symptoms and nervous system involvement. Meanwhile, the more objective support caregivers received, the higher the health-related quality of life of GD caregivers. Due to the nervous system involvement of type 2 GD [2], it is challenging to take care of patients with type 2 Gaucher 's disease. Health decision-makers and clinicians should take action to recover the life function of type 2 GD patients and give nursing suggestions to the caregivers of patients with Gaucher 's disease. The COI of GD patients with splenectomy surgery ($23,016.1 ± $16,832.3) is lower than GD patients without ($58,604.9 ± $73,187.1). The longer duration of the GD final confirmed diagnosis and the more challenging diagnosis, the higher COI of GD patients. Thus, the efficiency of clinical diagnosis should be improved. Simultaneously, clinicians need to judge the patient's condition accurately, and surgery is recommended if the indications are met. Therefore, patients with GD experience substantial costs and low health-related quality of life [71]; clinicians and society should help patients with Gaucher 's disease and their families as early as possible.

### Limitations

First, this study is limited primarily by the small sample size. Due to the rarity of GD, data collection was challenging. Second, families who voluntarily participate in the GD patient registry may represent a more compliant and motivated patient cohort in general. Thus, the findings may not be generalized to all. Thirdly, this study relied on responders’ recall of health service utilization and the unit costs to capture a more inclusive set of cost components usually not included in billing data, such as traffic costs, formal care costs, and productivity loss to caregivers. To enhance the accuracy of the estimates reported, patients with GD and caregivers were asked to complete questions regarding the average consumption amount and costs per resource in the past year. However, recall bias may have led to errors. Fourthly, utilization and corresponding costs from pharmaceutical use of prescription were not included in the survey. For pharmaceutical costs, we asked for the “annual purchase of imported/domestic medicines,” which could have resulted in an underestimation of this study’s GD’s financial burden. Hence, appraisal costs using data from health insurance is needed. Lastly, it was no possible to estimate informal care costs due to the form of the question we design to request this information, but we assessed the productivity losses of informal caregivers.

## Conclusions

Patients with GD often encountered the frustrating experience of high misdiagnosis rate, long-delayed diagnosis, substantial costs, and deteriorated health-related quality of life in China. For patients with GD, a rapid, precise diagnostic tool for earlier and timely definitive diagnosis of GD is urgently needed. Measures targeting improving the availability and affordability of orphan drug needs to be developed holistically in China. Furthermore, newborn screening for disorders that meet the criteria for population screening with reliable diagnostic tools should be explored. Besides, enhancing doctors’ ability in GD diagnosis and differential diagnosis, raising awareness of the early signs/symptoms or less severe manifestation to achieve earlier diagnosis and timely therapy of rare diseases, relieve caregivers’ burden, and provide much needed social support are essential. This research can facilitate greater societal awareness of rare diseases, help policymakers develop appropriate intervention programs, and inform healthcare-focused support schemes and policies for patients and their families.

## Supplementary Information

**Additional file 1.** Description/comparison of the characteristics, utilization of health service, perception of treatment, cost of illness, and scores of health-related quality of Gaucher disease (GD) families/caregivers/patients.

## Data Availability

Not applicable.

## Abbreviations

C-GDFEG: China Gaucher disease family exchange group
CI: Confidence interval
GD: Gaucher disease.
GDP: Gross domestic product
MCS: Mental Component Summary
PCS: Physical Component Summary
PSQI: Pittsburgh Sleep Quality Index
SD: Standard deviation
SF-36: Medical Outcomes Study 36-item Short Form
SSRS: Social Support Rating Scale
WHO: World Health Organization
ZBI: Zarit Burden Inventory
