# Supplementary material for: Economic burden and health related quality of life of ultra-rare Gaucher disease in China
Source: Orphanet J Rare Dis. 2021 Aug 11;16:358. doi: 10.1186/s13023-021-01963-6 (PMC8356434; doi:10.1186/s13023-021-01963-6)
Supplement: Supplementary file 1 — Additional file 1. Description/comparison of the characteristics, utilization of health service, perception of treatment, cost of illness, and scores of health-related quality of Gaucher disease (GD) families/caregivers/patients. [file 13023_2021_1963_MOESM1_ESM.docx]

**etable1 Characteristics of enrolled Gaucher disease families and caregivers.**

| **Family characteristics** | **Sample (n)** | **Proportion (%)** |
| --- | --- | --- |
| **Family history with GD** |  |  |
| No | 40 | 81.6 |
| Yes | 9 | 18.4 |
| **Current residence** |  |  |
| Urban | 19 | 38.8 |
| Rural | 30 | 61.2 |
| **Living on minimum subsistence allowance or relatives’ relief** |  |  |
| **No** | 32 | 65.3 |
| Yes | 17 | 34.7 |
| **Member with chronic disease** |  |  |
| No | 30 | 61.2 |
| Yes | 19 | 38.3 |
| **Member >60 years of age** |  |  |
| No | 7 | 14.3 |
| Yes | 42 | 85.7 |
| **Member <5 years of age** |  |  |
| No | 26 | 53.1 |
| Yes | 23 | 46.9 |
| **GD caregiver’s characteristics** |  |  |
| **Sex** |  |  |
| Male | 13 | 26.5 |
| Female | 36 | 73.5 |
| **Age** | 34.8±10.4 |  |
| **Education** |  |  |
| Primary School | 4 | 8.2 |
| Middle or high school | 30 | 61.2 |
| College | 15 | 30.6 |
| **Daily care time** |  |  |
| <6 h | 9 | 18.4 |
| 6-12 h | 17 | 34.7 |
| 12-18 h | 2 | 4.1 |
| 18-24 h | 21 | 42.9 |
| **Experience being caregiver** |  |  |
| No | 35 | 71.4 |
| Yes | 14 | 28.6 |
| **Main caregiver stopped working because of patients’ GD** |  |  |
| No | 10 | 20.4 |
| Yes | 39 | 79.6 |
| **Change of weekly working time because of patient’s GD** |  |  |
| No | 6 | 12.2 |
| Yes | 43 | 87.8 |
| **ZBI**  **Maximum scores, 88 points** |  |  |
| No | 30.6 |  |
| Yes | 69.4 |  |
| Mean±SD | 48.57±19.57 |  |

SD = standard deviation; ZBI = Zarit Burden Inventory-caregiver burden.

**etable 2 Descriptive analysis of the utilization of health service, including the diagnosis and treatment (n=49).**

| Items | Sample (n) | Proportion (%) | | |
| --- | --- | --- | --- | --- |
| **The original reason for visiting the doctors** |  |  |  |  |
| Osteopenia | 4 | 8.2 | 2.3 | 19.6 |
| Normocytic anemia | 18 | 36.7 | 23.4 | 50.2 |
| Dyskinesia | 6 | 12.2 | 3.1 | 21.4 |
| Obviously enlarged liver and spleen | 35 | 71.4 | 56.7 | 83.4 |
| **Top current symptoms** |  |  |  |  |
| Hepatosplenomegaly | 42 | 85.7 | 72.8 | 94.1 |
| Normocytic anemia | 36 | 73.5 | 58.9 | 85.1 |
| Bone pain or other bone symptoms | 17 | 34.7 | 21.7 | 49.7 |
| Growth retardation | 33 | 67.4 | 52.5 | 80.1 |
| Nervous system involvement | 10 | 20.4 | 10.2 | 34.3 |
| Gastrointestinal symptoms | 9 | 18.4 | 8.8 | 32.0 |
| **Types of medical institutions frequently visited** |  |  |  |  |
| Clinics/community health service centers | 1 | 2.0 | 0.1 | 11.5 |
| County-level medical institutions | 4 | 8.2 | 2.3 | 19.6 |
| Municipal medical institutions | 11 | 22.4 | 11.8 | 36.6 |
| Provincial medical institutions in the province | 10 | 20.4 | 10.2 | 34.3 |
| Provincial medical institutions in other provinces | 20 | 40.8 | 27.0 | 55.8 |
| None | 3 |  |  |  |
| **In the past year, outpatient visit for GD** |  |  |  |  |
| No | 11 | 22.4 | 11.8 | 36.6 |
| Yes | 38 | 77.6 | 63.4 | 88.2 |
| Mean±SD | 8.8±9.1 |  |  |  |
| **In the past year, inpatient visit for GD** |  |  |  |  |
| No | 12 | 24.5 | 13.3 | 38.9 |
| Yes | 37 | 75.5 | 61.1 | 86.7 |
| Mean±SD | 4.0±3.5 |  |  |  |
| **Traffic time** |  |  |  |  |
| <5 h | 24 | 49.0 | 34.4 | 63.7 |
| ≥5 h | 25 | 51.0 | 36.3 | 65.6 |
| **Number of hospitals visited for GD to confirm GD diagnosis** |  |  |  |  |
| Median | 3.0 |  |  |  |
| Mean±SD | 3.9±3.1 |  |  |  |
| Max | 20 |  |  |  |
| **Total cost for GD diagnosis** |  |  |  |  |
| Median | 7575.8 |  |  |  |
| Mean±SD | 15249.8±22854.3 |  | 8685.3 | 21814.4 |
| **Length of misdiagnosis (days)** |  |  |  |  |
| 0-60 | 14 | 28.6 | 16.6 | 43.3 |
| 60-120 | 9 | 18.4 | 8.8 | 32.0 |
| 120-180 | 4 | 8.2 | 2.3 | 19.6 |
| ≥180 | 22 | 44.9 | 30.7 | 59.8 |
| Mean±SD, years | 1.2±1.7 |  |  |  |
| **Duration of GD final confirmed diagnosed, years** |  |  |  |  |
| Mean±SD | 1.2±1.7 |  |  |  |
| Median | 0.4 |  |  |  |
| Max | 6.6 |  |  |  |
| **GD Misdiagnosis** |  |  |  |  |
| No | 20 | 40.8 | 27.0 | 55.8 |
| Yes | 29 | 59.2 | 44.2 | 73.0 |
| **Number of GD misdiagnosis** |  |  |  |  |
| Median | 2.0 |  |  |  |
| Mean±SD | 5.0±9.6 |  |  |  |
| **Before being correctly diagnosed with GD, (n=29)** |  |  |  |  |
| Unexplained splenomegaly | 19 | 65.5 | 48.2 | 82.8 |
| Hypersplenism | 13 | 44.8 | 26.5 | 64.3 |
| Hemophilia | 3 | 10.3 | 2.2 | 27.4 |
| **Therapy after diagnosis with GD** |  |  |  |  |
| No | 8 | 16.3 | 7.3 | 29.7 |
| Yes | 41 | 83.7 | 70.3 | 92.7 |
| **Surgery, splenectomy** |  |  |  |  |
| No | 39 | 79.6 | 65.7 | 89.8 |
| Yes | 10 | 20.4 | 10.2 | 34.3 |
| **Pharmacotherapy after diagnosis with GD** |  |  |  |  |
| No | 9 | 18.4 | 8.8 | 32.0 |
| Yes | 40 | 81.6 | 68.0 | 91.2 |
| **Purchased medicine at medical institutions, (n=40)** |  |  |  |  |
| No | 4 | 10.0 | 2.8 | 23.7 |
| Yes | 36 | 90.0 | 76.3 | 97.2 |
| **Purchased medicine at social pharmacy or overseas, (n=40)** |  |  |  |  |
| No | 9 | 22.5 | 10.8 | 38.5 |
| Yes | 31 | 77.5 | 61.6 | 89.2 |
| **Taking medication as prescribed, (n=40)** |  |  |  |  |
| No | 14 | 35.0 | 20.6 | 51.7 |
| Yes | 26 | 65.0 | 48.3 | 79.4 |

**SD = standard deviation; GD = Gaucher disease.**

**etable 3 Caregiver’ perception of Gaucher disease treatment (n=49).**

| Variables | Samples (n) | Proportion(%) | | |
| --- | --- | --- | --- | --- |
|  |  |  | Low limit | Upper limit |
| **Availability of therapeutic drugs** |  |  |  |  |
| Very insufficient | 19 | 38.8 | 25.2 | 53.8 |
| Insufficient | 9 | 18.4 | 8.8 | 32.0 |
| Medium | 16 | 32.7 | 20.0 | 47.5 |
| Sufficient | 5 | 10.2 | 3.4 | 22.2 |
| **Affordability of therapeutic drugs** |  |  |  |  |
| Very unaffordable | 7 | 14.3 | 5.9 | 27.2 |
| Unaffordable | 35 | 71.4 | 56.7 | 83.4 |
| Medium | 7 | 14.3 | 5.9 | 27.2 |
| **Knowledge of GD** |  |  |  |  |
| No | 48 | 98.0 |  |  |
| Yes | 1 | 2.0 |  |  |
| **The difficulty to get diagnosis information** |  |  |  |  |
| Difficult | 47 | 95.9 |  |  |
| Not difficult | 2 | 4.1 |  |  |
| **Feel of the difficult to treatment** |  |  |  |  |
| No | -- | -- |  |  |
| Yes | 49 | 100.0 |  |  |
| **Difficulties during GD treatment** |  |  |  |  |
| Far away from hospital |  |  |  |  |
| No | 12 | 25.0 | 13.6 | 39.6 |
| Yes | 37 | 75.0 | 60.4 | 86.4 |
| High treatment cost |  |  |  |  |
| No | 2 | 4.1 | 0.5 | 14.25 |
| Yes | 47 | 95.9 | 85.8 | 99.5 |
| **Extremely unconfident about future treatment** |  |  |  |  |
| No | 10 | 20.4 | 10.2 | 34.3 |
| Yes | 39 | 79.6 | 65.7 | 89.8 |

**etable 4 Comparison of cost of illness (COI) among GD patients.**

| **Characteristics** | **N=41** | **COI** | ***F*** | ***P*** |
| --- | --- | --- | --- | --- |
| **Sex** |  |  | 0.138 | 0.669 |
| Male | 20 | 54508.7±67268.6 |  |  |
| Female | 21 | 45559.0±65582.1 |  |  |
| **Age** |  |  | 0.1529 | 0.905 |
| <15 | 33 | 49.312.1±55971.4 |  |  |
| ≥15 | 8 | 52.451.9±101772.7 |  |  |
| **Types of Gaucher disease** |  |  | 0.476 | 0.707 |
| Type 1 | 19 | 60114.9±91443.7 |  |  |
| Type 2 | 5 | 61246.4±24233.5 |  |  |
| Type 3 | 3 | 40962.1±50896.8 |  |  |
| Not clear | 14 | 33972.3±25350.4 |  |  |
| **Outpatient visit** |  |  | 0.660 | 0.519 |
| No | 4 | 29471.1±32659.0 |  |  |
| Yes | 37 | 522135.9±68264.8 |  |  |
| **Inpatient visit** |  |  | 3.141 | 0.148 |
| No | 5 | 9858.2±5450.6 |  |  |
| Yes | 36 | 55489.5±68359.7 |  |  |
| **Surgery, splenectomy** |  |  | 4.634 | 0.017 |
| No | 31 | 58604.9±73187.1 |  |  |
| Yes | 10 | 23016.1±16832.3 |  |  |
| **Pharmacotherapy** |  |  | 1.194 | 0.523 |
| No | 5 | 32060.0±29347.7 |  |  |
| Yes | 36 | 52405.9±69190.1 |  |  |
| **Taking medication as prescribed** |  |  | 6.454 | 0.003 |
| No | 15 | 17277.7±19382.4 |  |  |
| Yes | 26 | 68759.5±75457.7 |  |  |
| **Disease course from symptom onset** |  | -- | 0.114 | 0.480 |
| **Duration of GD final confirmed diagnosed, years** |  |  | 0.458 | 0.003 |
| **The difficulty to get diagnose** |  |  | 0.388 | 0.012 |

**etable 5 Comparison of scores of health-related quality of life among GD patient.**

| **Characteristics** | **N=11** | **Hrqol** | ***F/r*** | ***P*** |
| --- | --- | --- | --- | --- |
| **Sex** |  |  | 1.680 | 0.554 |
| Male | 4 | 46.01±26.95 |  |  |
| Female | 7 | 38.61±13.81 |  |  |
| **Types of Gaucher disease** |  |  | -- | -- |
| Type 1 | 7 | 45.34±22.13 |  |  |
| Type 2 | -- | -- |  |  |
| Type 3 | 1 | 23.81 |  |  |
| Not clear | 3 | 37.81 |  |  |
| **Outpatient visit** |  |  | 3.720 | 0.094 |
| No | 4 | 28.86±4.30 |  |  |
| Yes | 7 | 48.40±20.15 |  |  |
| **Inpatient visit** |  |  | 3.073 | 0.924 |
| No | 4 | 42.07±28.60 |  |  |
| Yes | 7 | 40.86±12.94 |  |  |
| **Hepatosplenomegaly** |  |  | 3.030 | 0.916 |
| No | 4 | 42.15±29.07 |  |  |
| Yes | 7 | 40.81±12.41 |  |  |
| **Normocytic anemia** |  |  | 1.716 | 0.616 |
| No | 4 | 45.30±26.51 |  |  |
| Yes | 7 | 39.01±14.46 |  |  |
| **Bone pain or other bone symptoms** |  |  | 4.984 | 0.051 |
| No | 5 | 52.95±22.72 |  |  |
| Yes | 6 | 31.58±5.54 |  |  |
| **Nervous system involvement** |  |  | 1.314 | 0.291 |
| No | 9 | 44.22±19.35 |  |  |
| Yes | 2 | 28.13±6.63 |  |  |
| **Gastrointestinal symptoms** |  |  | 0.349 | 0.539 |
| No | 8 | 43.56±20.38 |  |  |
| Yes | 3 | 35.27±14.24 |  |  |
| **Daily care time** |  |  | 2.596 | 0.140 |
| <12 | 8 | 36.13±13.93 |  |  |
| ≥12 | 3 | 55.07±25.72 |  |  |
| **The difficulty to get diagnosis information** |  |  | 0.213 | 0.530 |
| **Health condition of GD patients** |  |  | 0.760 | 0.007 |
| **SSPS** |  |  | 0.410 | 0.210 |
| **Object support** |  |  | 0.216 | 0.524 |
| **Subjective support** |  |  | 0.430 | 0.186 |
| **Utilization of support** |  |  | 0.090 | 0.793 |

**etable 6 Comparison of scores of health-related quality of life among GD caregivers.**

| **Characteristics** | **N=49** | **Hrqol** | ***F/r*** | ***P*** |
| --- | --- | --- | --- | --- |
| Sex (patients) |  |  | 0.230 | 0.617 |
| Male | 25 | 47.90±20.42 |  |  |
| Female | 24 | 45.10±18.44 |  |  |
| Types of Gaucher disease (patients) |  |  | 3.528 | 0.022 |
| Type 1 | 20 | 51.29±20.31 |  |  |
| Type 2 | 5 | 23.38±11.62 |  |  |
| Type 3 | 4 | 40.01±16.65 |  |  |
| Not clear | 20 | 48.86±16.70 |  |  |
| Outpatient visit (patients) |  |  | 0.027 | 0.758 |
| No | 11 | 48.13±18.95 |  |  |
| Yes | 38 | 46.06±19.65 |  |  |
| Inpatient visit (patients) |  |  | 1.845 | 0.437 |
| No | 12 | 50.34±22.18 |  |  |
| Yes | 37 | 45.29±18.47 |  |  |
| Hepatosplenomegaly (patients) |  |  | 5.517 | 0.645 |
| No | 7 | 50.96±27.46 |  |  |
| Yes | 42 | 45.79±17.96 |  |  |
| Normocytic anemia (patients) |  |  | 2.771 | 0.520 |
| No | 13 | 49.52±23.41 |  |  |
| Yes | 36 | 45.44±17.87 |  |  |
| Bone pain or other bone symptoms (patients) |  |  | 3.409 | 0.070 |
| No | 32 | 50.17±20.70 |  |  |
| Yes | 17 | 39.67±14.61 |  |  |
| Nervous system involvement (patients) |  |  | 2.675 | 0.001 |
| No | 39 | 50.95±18.49 |  |  |
| Yes | 10 | 29.29±11.52 |  |  |
| Gastrointestinal symptoms (patients) |  |  | 2.574 | 0.788 |
| No | 40 | 46.17±20.50 |  |  |
| Yes | 9 | 48.12±13.69 |  |  |
| Daily care time (caregiver) |  |  | 5.610 | 0.053 |
| <12 | 26 | 41.40±15.02 |  |  |
| ≥12 | 23 | 52.33±22.18 |  |  |
| The difficulty to get diagnosis information |  |  | 0.212 | 0.143 |
| Health condition of GD patients |  |  | 0.465 | 0.001 |
| SSRS |  |  | 0.168 | 0.249 |
| Object support |  |  | 0.320 | 0.025 |
| Subjective support |  |  | 0.085 | 0.561 |
| Utilization of support |  |  | 0.060 | 0.682 |

**Cost calculations**

***Indirect cost***

The measurement of “***daily lost wages***” perceived by caregivers or patients was asked with a simple open question “How much is the fee due to the delay or absence of daily work.”

*The loss of productivity* = the daily lost wages of per caregiver * total days of lost wages annually[1,2]

**references**

1. BURQOL-RD Research Network, Angelis A, Kanavos P, López-Bastida J, Linertová R, Oliva-Moreno J, et al. Social/economic costs and health-related quality of life in patients with epidermolysis bullosa in Europe. Eur J Health Econ. 2016;17:31–42.

2. Maresova P, Lee S, Fadeyi OO, Kuca K. The social and economic burden on family caregivers for older adults in the Czech Republic. BMC Geriatr. 2020;20:171.
